# Supplementary material for: Regulation of the Gene for Alanine Racemase Modulates Amino Acid Metabolism with Consequent Alterations in Cell Wall Properties and Adhesive Capability in Brucella spp
Source: Int J Mol Sci. 2023 Nov 9;24(22):16145. doi: 10.3390/ijms242216145 (PMC10671322; doi:10.3390/ijms242216145)
Supplement: Supplementary file 1 [file ijms-24-16145-s001.zip › ijms-2630183-supplementary.pdf]

Table S1. Amino-acid composition of the ALR proteins.

Amino acid name	Number of each amino acid	The proportion of the total amino acids
Ala (A)	53	13.3%
Arg (R)	25	6.3%
Asn (N)	14	3.5%
Asp (D)	23	5.8%
Cys (C)	3	0.8%
Gln (Q)	8	2.0%
Glu (E)	17	4.3%
Gly (G)	36	9.0%
His (H)	7	1.8%
Ile (I)	20	5.0%
Leu (L)	51	12.8%
Lys (K)	17	4.3%
Met (M)	6	1.5%
Phe (F)	14	3.5%
Pro (P)	20	5.0%
Ser (S)	20	5.0%
Thr (T)	25	6.3%
Trp (W)	1	0.3%
Tyr (Y)	14	3.5%
Val (V)	25	6.3%

Table S2. Chemically defined medium, CDM

Composition	Content(μg/ml)
L-Tryptophan (L-Trp)	25
L-Tyrosine (L-Tyr)	25
L-Phenylalanine (L-Phe)	25
L-Cysteine (L-Cys)	5
L-Hstidine (L-His)	100
L-Methionine (L-Met)	50
L-Glutamic acid (L-Glu)	3000
Glycine (Gly)	100
L-Proline (L-Pro)	100
L-Threonine (L-Thr)	25
L-Valine (L-Val)	100
L-Leucine (L-Leu)	50
L-Isoleucine (L-Ile)	50
DL-Alanine (DL-Ala)	100
L-Arginine (L-Arg)	100
L-Lysine (L-Lys)	100
L-Aspartic acid (L-Asp)	200
L-Serine (L-Ser)	25
Glucose	4000
Magnesium sulfate	100
Sodium thiosulfate	100
NaCl	7500
Dipotassium hydrogen phosphate	1000
Thiamin	0.2
Nicotinic acid	0.2
Pantothenate	0.04
Biotin	0.001
Fe^{++}	0.1
Mn^{++}	0.1

Use NaOH to adjust pH to 6.8-7.0, add water to 1L, use 0.22μM filter to remove bacteria, and store at 4°C.

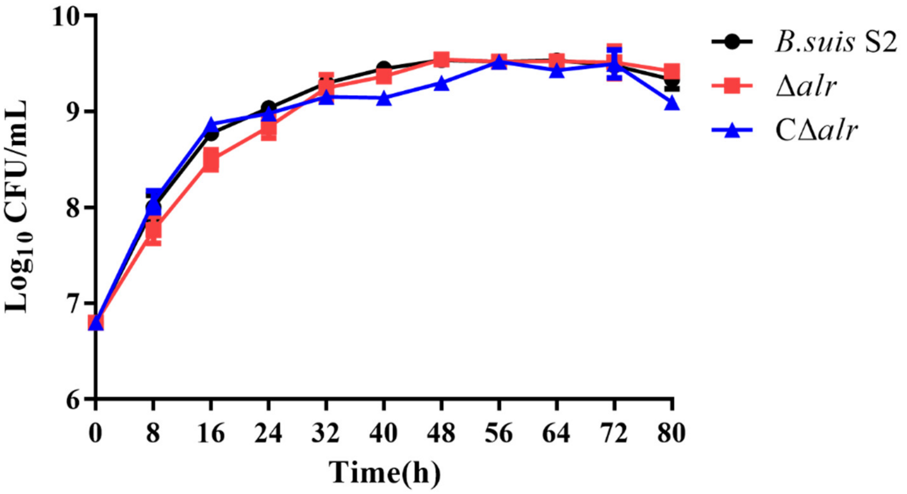

Figure S1. Growth curves of three *Brucella* strains. The Y-axis represents Colony-Forming Units (CFU), and the X-axis represents the incubation time.
